# Supplementary figures and images for: Assembly of the salt-secreting mangrove Avicennia rumphiana
Source: PLoS One. 2025 Feb 5;20(2):e0318091. doi: 10.1371/journal.pone.0318091 (PMC11798505; doi:10.1371/journal.pone.0318091)

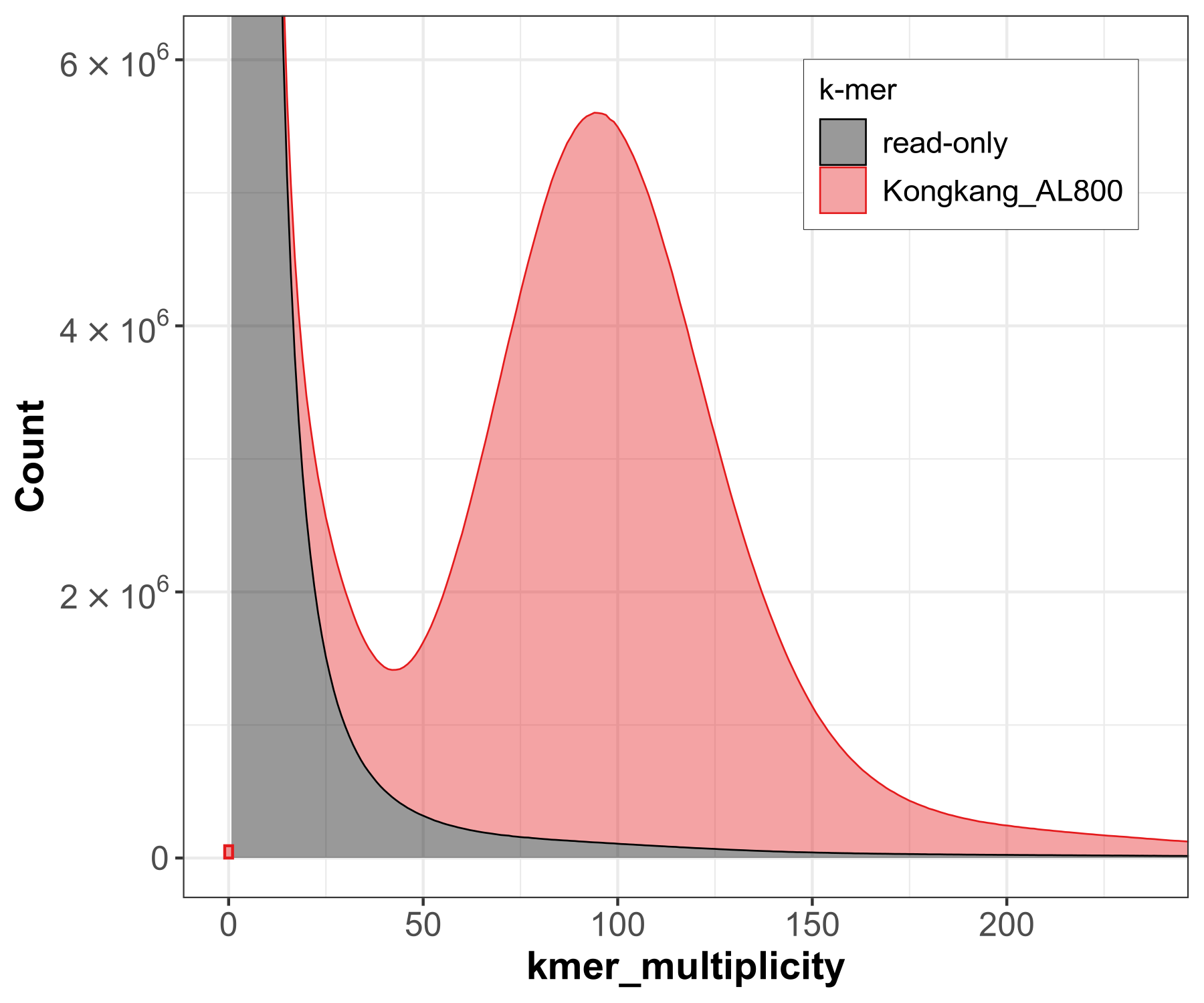

Supplement: S1 Fig — A single well-defined peak indicates a complete assembly with minimal redundancy. (PNG) [file pone.0318091.s001.png]

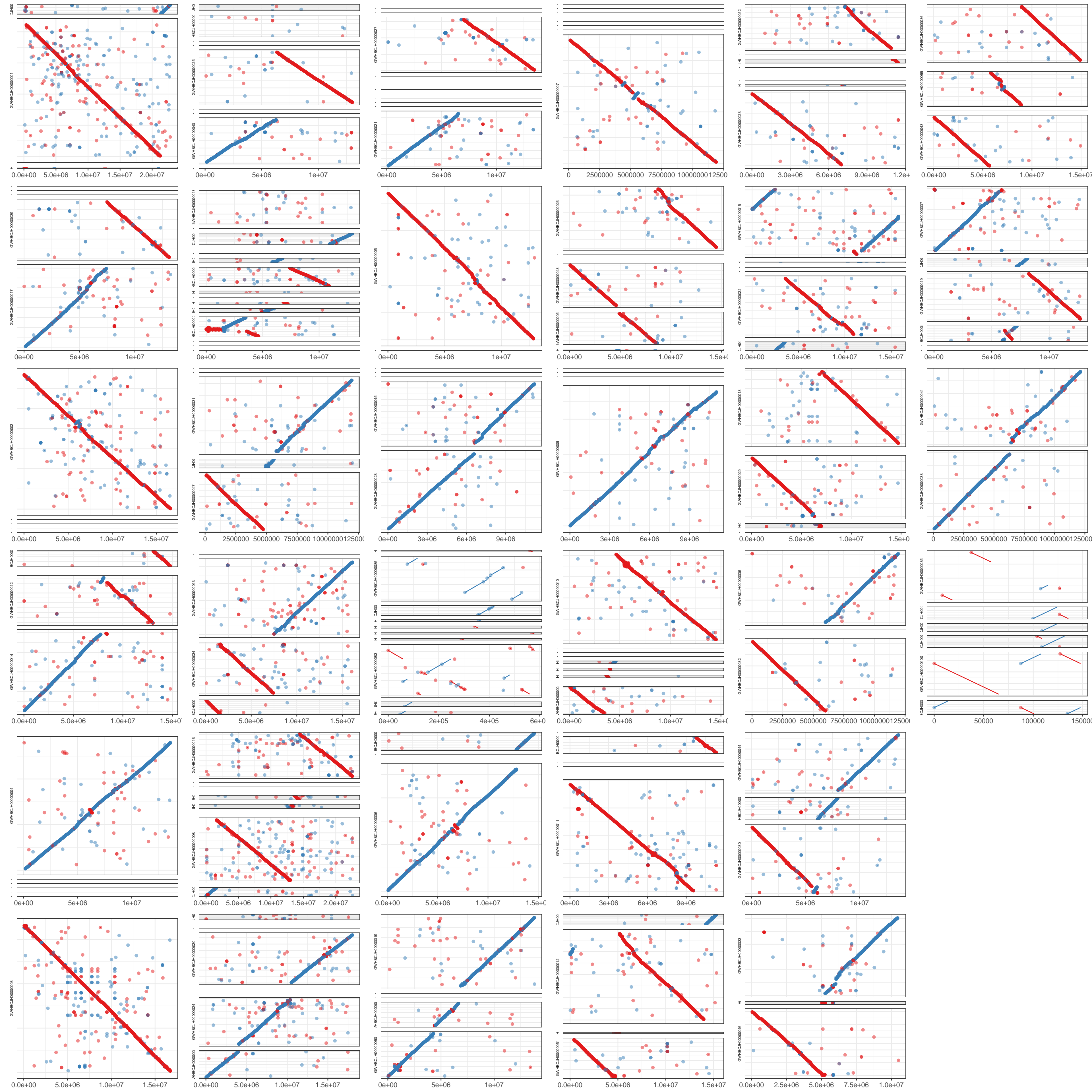

Supplement: S2 Fig — Cases where multiple A. rumphiana contigs map to a single A. marina chromosome appear as stacked boxes where the length of the y-axis is proportional to the amount of sequence that maps, resulting in small regions appearring as individual lines. Blue dots indicate direct sequence alignment, while red dots indicate inverted sequence alignment. (PDF) [file pone.0318091.s002.pdf]

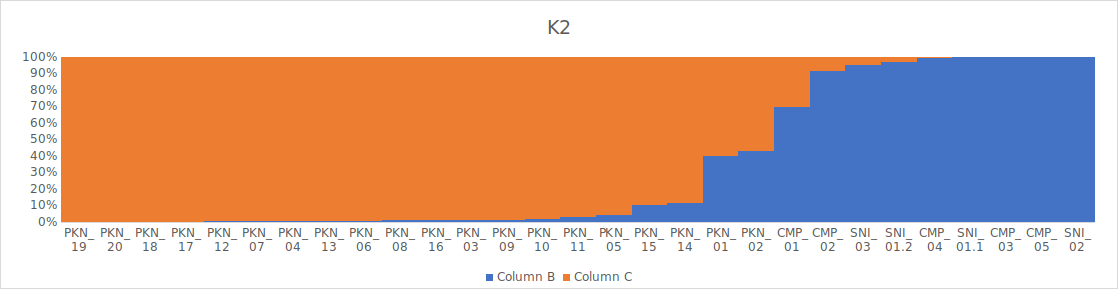

Supplement: S3 Fig — (TIF) [file pone.0318091.s003.tif]
