# Supplementary material for: Assembly of the salt-secreting mangrove Avicennia rumphiana
Source: PLoS One. 2025 Feb 5;20(2):e0318091. doi: 10.1371/journal.pone.0318091 (PMC11798505; doi:10.1371/journal.pone.0318091)
Supplement: Attachment — Submitted filename: Comments to the Author.docx [file pone.0318091.s008.docx]

**Comments to the author:**

This paper described a mangrove species *A. rumphiana* genome assembly and identification of salt genes. In general, there are some useful data generated in this paper. However, the mechanisms underlying needs to be explored. For example, we would like know which genes respond to salinity environment, which provides valuable insight into the molecular mechanisms of salinity tolerance in *A. rumphiana.* Some concerns needed to be addressed before further consideration.

1. L95-97: The sequenced data should be uploaded to public platform, such as NCBI or NGDC (https://ngdc.cncb.ac.cn/gsa). In this study, authors used short sequenced reads (150bp) to do *de novo* assembly of *A. rumphiana* genome*.* However, the repetitive genome regions are often difficult to assembly using short-read sequencing technologies. The published *A. rumphiana* assembly (GWHBCJH00000000) also revealed that there are more than 50% repetitive sequence in the whole genome. Therefore, it’s required to add new sequenced data, such as PacBio or ONT Long-read data, even Hic-data.
2. L103-105: The authors present a chromosome-level scaffolds of *A. rumphiana* using a reference-guided approach. Why did you choose *A. marina* as reference genome for scaffolding? According to the phylogenetic tree from He *et al*. study (paper: Evolution of coastal forests based on a full set of mangrove genomes), *Avicennia alba* is closer mangrove species to *A. rumphiana* compared to *A. marina.* Additionally, several reference genomes of *A. marina* are publicly available, which *A. marina* genome is better for your genome assembly of *A. rumphiana*, why?
3. L115-122: The genome annotation was not correct. To predict genes accurately in *A. rumphiana* assembly, the genome should be masked for repeats before genome annotation. This will avoid the prediction of false positive gene structures in repetitive and low complexity regions. In addition, the authors only predicted genes using ab initio prediction program Augustus. RNA-seq data from different tissues of *A. rumphiana* should be used to improve gene prediction accuracy.
4. L186-L187: The expansion and contraction of gene families served as a key source for plants to acquire adaptive function. For this purpose, GO and KEGG enrichment analysis need to be performed.
5. L198-205: The sequenced data should be uploaded to public platform, such as NCBI or NGDC (https://ngdc.cncb.ac.cn/gsa). After sequencing, a quality control of raw data was required to perform and low-quality reads need to be removed. In addition, detailed parameters, filtering condition of software for reads alignment and variants calling are not clear.
6. L227-228 and L243-244: The authors revealed that approximately 68.7% of the *A. rumphiana* genome sequence can be aligned to *A. marina* sequence, and 91.2% of *A. rumphiana* can be mapped to the published assembly (GWHBCJH00000000). This made me confused. Why did not you choose published assembly (GWHBCJH00000000) as your reference genome for scaffolding? Additionally, the assembly completeness of genome was need to be evaluated using BUSCO software and LTR Assembly Index (LAI) metric.
7. L383 and L290: The authors performed the population analysis and identification of salt genes, however, no further analysis was done for data mining, such as genetic diversity, genotype-environment association analysis, *etc*. The main problem is that it lacks innovation and new insight.
