# Supplementary material for: Assembly of the salt-secreting mangrove Avicennia rumphiana
Source: PLoS One. 2025 Feb 5;20(2):e0318091. doi: 10.1371/journal.pone.0318091 (PMC11798505; doi:10.1371/journal.pone.0318091)
Supplement: Attachment — Submitted filename: Response to reviewers R2.docx [file pone.0318091.s009.docx]

**Comments to the author:**

This paper described a mangrove species *A. rumphiana* genome assembly and identification of salt genes. In general, there are some useful data generated in this paper. However, the mechanisms underlying needs to be explored. For example, we would like know which genes respond to salinity environment, which provides valuable insight into the molecular mechanisms of salinity tolerance in *A. rumphiana.* Some concerns needed to be addressed before further consideration.

1. L95-97: The sequenced data should be uploaded to public platform, such as NCBI or NGDC (https://ngdc.cncb.ac.cn/gsa). In this study, authors used short sequenced reads (150bp) to do *de novo* assembly of *A. rumphiana* genome*.* However, the repetitive genome regions are often difficult to assembly using short-read sequencing technologies. The published *A. rumphiana* assembly (GWHBCJH00000000) also revealed that there are more than 50% repetitive sequence in the whole genome. Therefore, it’s required to add new sequenced data, such as PacBio or ONT Long-read data, even Hic-data.

**Response**: The raw data should have been available at the time of review using the accession in the ‘Accession codes’ section at the end of the manuscript, upon checking, it is currently available at the end of the list of raw sequence data (SRX24708924).

10x genomics is not just short read sequencing, it uses emulsion PCR to perform barcoded sequencing of high molecular weight DNA (https://ucdavis-bioinformatics-training.github.io/2018-Dec-Genome-Assembly/10x-supernova/10x-supernova.html) to achieve a level of information similar to, or even in some cases, better than current long read technologies. While we would like to add Hi-C, we do not have the funding for it and must publish what we have. We think we have provided sufficient evidence to support use of reference based scaffolding instead.

2. L103-105: The authors present a chromosome-level scaffolds of *A. rumphiana* using a reference-guided approach. Why did you choose *A. marina* as reference genome for scaffolding? According to the phylogenetic tree from He *et al*. study (paper: Evolution of coastal forests based on a full set of mangrove genomes), *Avicennia alba* is closer mangrove species to *A. rumphiana* compared to *A. marina.* Additionally, several reference genomes of *A. marina* are publicly available, which *A. marina* genome is better for your genome assembly of *A. rumphiana*, why?

**Response**: We added the sentence “Considering only species with a chromosome-level genome assembly available, *A. marina* is the most closely related species to *A. rumphiana*.” to the introduction L63-65. A. alba was only assembled at the contig level.

We chose the assembly from Natarajan et al since it was done using long PacBio reads and had the annotation for salt genes available. However, there is no indication as to which, if any, of the 3 genome assemblies is better. We added the accession number of the A. marina assembly that was used L101.

3. L115-122: The genome annotation was not correct. To predict genes accurately in *A. rumphiana* assembly, the genome should be masked for repeats before genome annotation. This will avoid the prediction of false positive gene structures in repetitive and low complexity regions. In addition, the authors only predicted genes using ab initio prediction program Augustus. RNA-seq data from different tissues of *A. rumphiana* should be used to improve gene prediction accuracy.

**Response**: The annotation was performed on the masked genome, the wording has been corrected to reflect this L118. Annotation was performed using EvidenceModeller, which combined gene prediction and protein sequence from related species in the database. We do not have RNA-seq data due to budget constraints, but it would be slightly better if we did.

4. L186-L187: The expansion and contraction of gene families served as a key source for plants to acquire adaptive function. For this purpose, GO and KEGG enrichment analysis need to be performed.

**Response**: We performed GO analysis on the group of expanded genes and the group of contracted genes. KEGG pathway data was sparse for these two groups, so we decided that the results would not be reliable and focused only on GO analysis. This was added to the materials and methods, line 192-196, and discussed in lines 378-385.

5. L198-205: The sequenced data should be uploaded to public platform, such as NCBI or NGDC (https://ngdc.cncb.ac.cn/gsa). After sequencing, a quality control of raw data was required to perform and low-quality reads need to be removed. In addition, detailed parameters, filtering condition of software for reads alignment and variants calling are not clear.

**Response**: The data is available on NCBI (see comment 1). WGS read quality was checked and found to be good, but reads were not filtered prior to using the Supernova assembly software because of the barcoded nature of the reads. We added ‘with default parameters’ to the appropriate sections. Filtering was also not performed for the population samples as the mapping software excludes low quality alignments by default.

6. L227-228 and L243-244: The authors revealed that approximately 68.7% of the *A. rumphiana* genome sequence can be aligned to *A. marina* sequence, and 91.2% of *A. rumphiana* can be mapped to the published assembly (GWHBCJH00000000). This made me confused. Why did not you choose published assembly (GWHBCJH00000000) as your reference genome for scaffolding? Additionally, the assembly completeness of genome was need to be evaluated using BUSCO software and LTR Assembly Index (LAI) metric.

**Response**: The published assembly is not at the chromosome level, although we did show that some of their contigs were whole chromosomes. We did perform BUSCO L126-128 & L305-307. We performed Merqury analysis L103-105 & L227-229, which gives a reference free genome assembly quality analysis. Adding another seems excessive.

7. L383 and L290: The authors performed the population analysis and identification of salt genes, however, no further analysis was done for data mining, such as genetic diversity, genotype-environment association analysis, *etc*. The main problem is that it lacks innovation and new insight.

**Response**: The PCA, PIC and STRUCTURE analysis reports on genetic diversity to the full extent that the data allows. We did not collect and data that can be used to identify any kind of genotype-environment associations, that is outside the scope of this study.

Reviewer #3: 1) Just a comment regarding the sentence (abstract):
“...Sequence comparison showed that 68.7% of the A. rumphiana genome aligned to A. marina sequence, covering 72% of the A. marina genome at an average nucleotide identity of 87.7%, suggesting A. marina is suitable for reference based scaffolding of the A. rumphiana contigs...”
*I believe authors should explain better this fact, in face of evolution features.

**Response**: We added that this shows A. marina is closely related to A. rumphiana in the abstract. We added some discussion about this to the results and discussion section L261-264

2) *for ecological meaning (population), I believe authors should explain better how health level exist, regarding of "...genetic variation exists in the population..." and the relationship with "...which is the least severe status in the ‘threatened' category…”

**Response**: We added the sentence “A population with a low level of genetic diversity can be at increased risk of collapse from mechanisms such as inbreeding depression and reduced adaptability to biotic or abiotic stresses.” to L 405-407
